# Cell ID and Timing Estimation Techniques for Underwater Acoustic Cellular Systems in High-Doppler Environments

**DOI:** 10.3390/s20154147

**Published:** 2020-07-26

**Authors:** Muhammad Asim, Mohammed Saquib Khan, Tae Ho Im, Yong Soo Cho

**Affiliations:** 1 Department of Electrical and Electronics Engineering, Chung-Ang University, Seoul 156-756, Korea; muhammad3428@gmail.com (M.A.); snhk02@gmail.com (M.S.K.); 2 Department of Oceanic IT Engineering, Hoseo University, Asan-si 336-795, Korea; taehoim@hoseo.edu

**Keywords:** underwater acoustic cellular system, cell ID estimation, timing estimation, Doppler shift

## Abstract

In an underwater acoustic cellular (UAC) system, underwater equipment or sensor nodes need to detect the identity of an underwater base station (UBS) and synchronise it with a serving UBS. It is known that, in an underwater acoustic channel, the temporal variability of the ocean coupled with the low speed of sound in water may induce a significant Doppler shift. In this paper, two different types of cell search techniques (CSTs) are proposed to detect the cell ID and correct timing of the UBS in UAC systems with a Doppler shift: CST based on linear frequency modulation with full bandwidth in the time domain (LFM-FT) and CST based on linear frequency modulation in the frequency domain (LFM-FF). The performances (auto-correlation, cross-correlation, ambiguity function, and cross ambiguity function) of the proposed techniques are analysed and compared with simulation results. It is demonstrated by simulation that the proposed techniques perform better than previous techniques in both AWGN and multipath channels when a Doppler shift exists. It is also shown that the LFM-FF-CST achieves the best performance in the presence of a Doppler shift and is suitable for mobile UAC systems.

## 1. Introduction

With the tremendous scientific and technological advances over recent decades, a wide range of underwater exploration applications have emerged. The scientific exploration spans multiple disciplines, such as coastal surveillance, disaster prevention, oil and gas exploration, and environmental monitoring. With the application of data collection such as environmental/security data, and real-time videos, it may require to deploy autonomous underwater vehicles (AUVs) or sea gliders in the ocean over a long duration [1,2,3,4,5,6,7,8]. To establish a communication link between nodes in underwater networks, several communication media such as cables, electromagnetic waves, optical waves, and acoustic waves have been used. In underwater wireless communication systems, acoustic waves are extensively used since acoustic waves propagate well in underwater and can traverse large distances. However, compared with terrestrial radio frequency communication systems, underwater channels are severely affected by several transmission losses (absorptive loss, geometric spreading, and scattering loss). The acoustic wave propagates through multipath channel caused by the reflections on the surface of the ocean and refractions between different layers due to the depth-varying sound speed. The temporal variability of the ocean combined with the low sound speed in water may induce significant Doppler shifts [9,10]. In underwater communication, the low frequency band is normally used because the attenuation of acoustic waves increases with frequency.

Spatial frequency reuse was considered for large area coverage in bandwidth-limited underwater acoustic networks [11]. Based on the acoustic propagation laws (dependence of path loss on both distance and frequency), the analysis offers a relatively simple tool for the design of autonomous underwater systems based on cellular network architectures. In [12], a channel sharing protocol for underwater wireless sensor networks based on a cellular type of architecture was proposed by leveraging the long delays and signal absorption in the underwater channel, resulting in an increased efficiency of bandwidth utilisation. In [13], the cell size and frequency reuse pattern are investigated to support a desired number of users operating over a given area in an underwater acoustic cellular (UAC) network. In [14], the concept of frequency reuse is investigated to increase both the coverage and capacity in a UAC system. In [15], it is shown that the capacity of a UAC system based on orthogonal frequency division multiplexing (OFDM) is higher than that of a single carrier system. A code-division multiple access (CDMA)-based underwater acoustic cellular system and cellular underwater wireless optical CDMA system were proposed in [4,16,17,18].

In a UAC system, the user equipment (UE) should perform downlink (DL) synchronisation and cell searching using the received preamble to establish a communication link. The preamble is normally transmitted from the UBS in the DL header for synchronisation and cell search. Many different types of sequences have been developed for synchronisation since synchronisation requirements vary for each application. The Barker sequence was used for preamble design in the underwater acoustic channel. However, it has some limitations because it is usually modulated with tone carrier, which is easily distorted in underwater channels [19]. An M sequence has been widely used for synchronisation in terrestrial communication because it exhibits a good autocorrelation property. A complex polyphase sequence, Zadoff-chu sequence (ZCS), has been widely used for synchronisation in OFDM systems because it has an ideal autocorrelation and small cross-correlation properties. However, the ZCS is sensitive to Doppler shift, which is unavoidable in UAC systems [20]. In an underwater acoustic channel, chirp signals, such as linear frequency modulation (LFM) and hyperbolic frequency modulation (HFM), are often used for signal detection due to their robustness against Doppler shift [21]. Although LFM signals have been widely used in radar and sonar surveillance applications, they have not yet been used for the design of preamble in cellular networks. In cellular networks, a large number of preambles are required to distinguish the preambles transmitted from different base stations at the mobile station.

For underwater acoustic systems, many synchronisation techniques such as signal detection, timing/frequency acquisition, and Doppler scale estimation/compensation have been developed [21]. However, there is a dearth of research on the cell search technique (CST) for estimating the physical cell identity (CID) of the UBS. The conventional terrestrial cellular system’s CSTs cannot be applied to the UAC system because the slow propagation of acoustic waves produces a large Doppler spread and/or shift. In [22], two different types of CST were proposed for the UAC system, i.e., ZCS-based CST (ZCS-CST) and LFM-based CST (LFM-CST). Since ZCS-CST is based on the ZCS, it can be considered as a modified version of the CST in the LTE systems. In LFM-CST, by changing the parameters of the LFM signal (frequency sweeping parameter and starting frequency), multiple LFM preambles are generated to map the CIDs to these preambles. The LFM-CST was seen to perform better than the ZCS-CST when a Doppler shift exists. However, in the LFM-CST, full bandwidth cannot be assigned to all preambles since the CID is mapped to its frequency sweeping parameter. Thus, in the LFM-CST, system bandwidth cannot be optimally utilised. The CID detection probability will decrease when the preambles with a small value of frequency sweeping parameter are used. The range resolution decreases as the value of the frequency sweeping parameter decreases. Furthermore, the correlation peak in the LFM signal is shifted from the correct position, with an increase in the Doppler shift.

In order to overcome these limitations, two different types of CST are proposed in this paper: CST based on linear frequency modulation with full bandwidth in the time domain (LFM-FT) and CST based on linear frequency modulation in the frequency domain (LFM-FF). In both the LFM-FT-CST and LFM-FF-CST, multiple LFM preambles are generated by changing the frequency sweeping parameters of the LFM signal as in the previous LFM-CST. However, unlike the LFM-CST, full bandwidth is used for all the preambles in the LFM-FT-CST. In the LFM-FT-CST, the preambles are directly generated in the time domain with two different frequency sweeping parameters, one for each of the two halves of the preamble duration. In the LFM-FF-CST, the LFM waveforms are generated in the frequency domain with different frequency sweeping parameter and are converted into time-domain signal via an inverse discrete Fourier transform (IDFT). All the preambles in both the LFM-FT-CST and LFM-FF-CST have the same power.

The rest of this paper is organised as follows. Two previous cell search techniques for UAC systems are summarised in Section 2. Two cell search techniques (LFM-FT-CST and LFM-FF-CST) are proposed for UAC systems in high Doppler environments in Section 3. The auto-correlation and cross-correlation functions of LFM-FT-CST are analysed and compared with the simulation results. The ambiguity function (AF) and cross ambiguity function (CAF) of LFM-FF-CST are derived and compared with the simulation results. In Section 4, the performances of the proposed CSTs are evaluated for AWGN and multipath channels using a Bellhop channel simulator. The conclusions are drawn in Section 5.

## 2. Preliminaries

**Figure 1:**
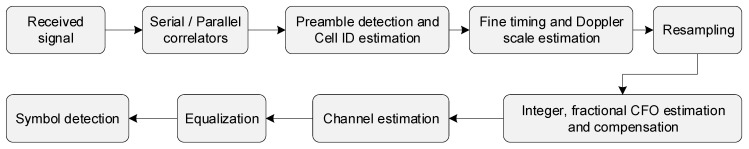
Typical receiver processing modules for OFDM-based underwater communication systems.

Figure 1 depicts the typical receiver processing modules in OFDM-based underwater communication systems. One of the most important tasks of the receiver is to detect the arrival of the useful signals from the transmitter because underwater acoustic channels are known to be one of the most challenging channels for communication. An LFM waveform is usually used for preamble detection because it is known to be robust to the Doppler effect. As illustrated in Figure 1, the received signal carrying a detection preamble is correlated with the bank of locally generated preambles to perform signal detection and obtain coarse timing. In the UAC system, the UE should also detect the CID of the serving UBS. Once the preamble is detected, the receiver starts recording the waveform from the detected timing instant. The receiver estimates the fine timing by using the received synchronisation preamble and estimates the Doppler scale factor because the transmitted signal is usually dilated or compressed at the receiver end. Using the estimated Doppler scale factor, the Doppler scaling effect is compensated by resampling the received signal. The estimation and compensation of integer and fractional carrier frequency offsets (CFOs) are performed using the repetitive patterns of the synchronisation preamble. The transmitted data are recovered with a frequency-domain equalizer using the pilots inserted in each OFDM symbol. In this paper, we will focus on the CST for estimating the CID and correct timing of the UBS because many synchronisation techniques for underwater acoustic channels are already available.

Next, the previous CSTs (ZCS-CST and LFM-CST) developed for a UAC are summarised [22]. In the ZCS-CST, cell searching is performed independently of the preamble detection. After completing signal detection using the LFM waveform, cell searching is performed using the received ZCS. In this technique, like the LTE system, the root index of the ZCS is used to map the CID. In the absence of Doppler frequency shift, due to the good correlation property of ZCS, a high CID detection probability is achieved regardless of AWGN and multipath channels. However, when Doppler shift exists, the detection probability of the ZCS-CST decreases significantly. In LFM-CST, the received LFM waveform is used to perform cell search in conjunction with signal detection and coarse timing detection. Here, multiple LFM preambles are generated to estimate different CIDs from the received LFM waveform (at sensor node). The CID of the UBS is mapped to the starting frequency and frequency sweeping parameters of the LFM waveform. The received signal is then correlated with a bank of locally generated LFM waveforms with different parameters for signal detection and CID estimation. The LFM-CST shows better performance (higher CID detection probability) than the ZCS-CST in the presence of a Doppler shift, because the LFM waveform is insensitive to the Doppler shift.

However, the LFM-CST has the following weakness. In the LFM-CST, multiple LFM preambles are generated to distinguish a serving UBS from adjacent UBSs. The multiple LFM preambles are generated by changing the frequency sweeping parameter of the LFM waveform. The CID of a UBS is mapped to the frequency sweeping parameter of the LFM waveform. Thus, the UBSs are assigned different frequency sweeping parameters, implying that different bandwidths are used for the UBSs. Full bandwidth cannot be assigned to all the UBSs. Subsequently, the CID detection probability will vary depending on the frequency sweeping parameter assigned to the UBS. The detection probability will be the highest when the frequency sweeping parameter is equal to its system bandwidth, because the LFM waveform is qualified as a pulse compression waveform when the BT product (time-bandwidth product) is much greater than one. The detection probability will decrease as the value of frequency sweeping parameter decreases. The range resolution also decreases with a decrease in the value of the frequency sweeping parameter.

The LFM waveform is known as a Doppler-insensitive waveform because it can produce a significant output peak for a wider range of Doppler shift. However, when a Doppler shift exists, its peak will not occur at the correct timing. It will be shifted by an amount proportional to the Doppler shift. The correlation peak is shifted further from the correct position as the Doppler shift increases. Thus, the LFM-CST may produce incorrect coarse timing in the presence of a large Doppler shift.

## 3. Proposed Cell Search Techniques for UAC Systems

In this section, we propose two different CSTs for UAC systems to overcome the limitations of the LFM-CST. In this study, it is assumed that the bandwidth assigned to each cell is *W* Hz. The frequency reuse factor in a UAC system is 1. It is also assumed that preambles are transmitted from UBSs to facilitate synchronisation and cell searching. The UE receives preambles from *N* neighbouring UBSs.

### 3.1. Cell Search Technique Based on LFM with Full Bandwidth in Time Domain (LFM-FT)

In the LFM-FT-CST, a preamble is constructed with two different frequency sweeping parameters in the signal duration. A frequency sweeping parameter is used in the first half of the signal duration and the complementary frequency sweeping parameter is used in the second half of the signal duration. The starting frequency for the first half of the signal duration is set to either 0 or *W* Hz. When the starting frequency of the first half is 0/W Hz, the starting frequency of the second half is selected such that the instantaneous frequency ends in W/0 Hz. In this case, positive/negative frequency sweeping parameters are used for both the first and second halves. Thus, the preamble always uses full bandwidth (*W* Hz), regardless of its selection of frequency sweeping parameter. In the LFM-FT-CST, the preamble carrying CID *b* of *b*th UBS with a symbol duration $T_{b}$ is defined in the time domain as

(1) $$\begin{array}{l} x_{b,1}^{\mathrm{LFM}\_\mathrm{FT}}(t)=e^{j\pi (2f_{b,1}t+\frac{\beta_{b,1}}{T_{b}/2}t^{2})}\;\;0\leq t\leq \frac{T_{b}}{2}\\ x_{b,2}^{\mathrm{LFM}\_\mathrm{FT}}(t)=e^{j\pi (2f_{b,2}(t-\frac{T_{b}}{2})+\frac{\beta_{b,2}}{T_{b}/2}{\left(t-\frac{T_{b}}{2}\right)}^{2})}\;\;\frac{T_{b}}{2}\leq t\leq T_{b} \end{array}$$

where $x_{b,1}^{\mathrm{LFM}\_\mathrm{FT}}(t)$ and $x_{b,2}^{\mathrm{LFM}\_\mathrm{FT}}(t)$ represent the first and second half of the preamble, respectively. In addition, $f_{b,1}$ and $\beta_{b,1}$ denote the starting frequency and frequency sweeping parameter for the first half of the preamble, respectively. $f_{b,2}$ and $\beta_{b,2}$ denote the starting frequency and frequency sweeping parameter for the second half of the preamble, respectively. $f_{b,2}$ is equal to $\left(f_{b,1}+\beta_{b,1}\right)$ and $\beta_{b,2}$ is less than or equal $\left(W-f_{b,2}\right)$. In the LFM-FT-CST, a combination of $\left(f_{b,1},\beta_{b,1}\right)$ is used to map the CID of UBS. The parameters $\left(f_{b,2},\beta_{b,2}\right)$ are determined such that the instantaneous frequency ends in *W* or 0 Hz. The auto-correlation function of LFM-FT signal can be derived as follows:

(2) $$\begin{array}{cc} \rho_{b}^{\mathrm{LFM}\_\mathrm{FT}}(\tau ,\varepsilon )= & \int_{-\infty }^{\infty }x_{b,1}^{\mathrm{LFM}\_\mathrm{FT}}(s)x_{b,1}^{\mathrm{LFM}\_\mathrm{FT}}{\left(s-\tau \right)}^{*}e^{j2\pi \varepsilon s}ds+\\ & \int_{-\infty }^{\infty }x_{b,2}^{\mathrm{LFM}\_\mathrm{FT}}(s-\frac{T_{b}}{2})x_{b,2}^{\mathrm{LFM}\_\mathrm{FT}}{\left(s-\frac{T_{b}}{2}-\tau \right)}^{*}e^{j2\pi \varepsilon s}ds\\ = & \int_{-\infty }^{\infty }e^{j\pi (2f_{b,1}s+\frac{\beta_{b,1}}{T_{b}/2}s^{2})}e^{-j\pi (2f_{b,1}(s-\tau )+\frac{\beta_{b,1}}{T_{b}/2}{\left(s-\tau \right)}^{2})}e^{j2\pi \varepsilon s}ds+\\ & \int_{-\infty }^{\infty }e^{j\pi (2(f_{b,1}+\beta_{b,1})(s-\frac{T_{b}}{2})+\frac{W-f_{b,2}}{T_{b}/2}{\left(s-\frac{T_{b}}{2}\right)}^{2})}\times \\ & e^{-j\pi (2(f_{b,1}+\beta_{b,1})(s-\frac{T_{b}}{2}-\tau )+\frac{W-f_{b,2}}{T_{b}/2}{\left(s-\frac{T_{b}}{2}-\tau \right)}^{2})}e^{j2\pi \varepsilon s}ds\\ = & e^{j\pi (2f_{b,1}+\varepsilon )\tau }(T_{b}-\tau )\mathrm{sinc}(\varepsilon \frac{T_{b}}{2}+\beta_{b,1}\tau )(1-\frac{\tau }{\left|T_{b}\right|})+\\ & e^{j\pi ((f_{b,1}+\beta_{b,1})-2(W-f_{b,2})+\varepsilon )\tau }(T_{b}-\tau )\mathrm{sinc}(\varepsilon T_{b}+2(W-f_{b,2})\tau )(1-\frac{\tau }{\left|T_{b}\right|}) \end{array}$$

where ϵ and τ denote the Doppler shift and time difference $\left(-T_{b}\leq \tau \leq T_{b}\right)$, respectively. When ϵ = 0, (2) becomes

(3) $$\begin{array}{cc} \rho_{b}^{\mathrm{LFM}\_\mathrm{FT}}(\tau ,0) & =e^{j\pi (2f_{b,1})\tau }(T_{b}-\tau )\mathrm{sinc}(\beta_{b,1}\tau )(1-\frac{\tau }{\left|T_{b}\right|})+\\ & e^{j\pi (\left(f_{b,1}+\beta_{b,1})-2(W-f_{b,2}\right))\tau }(T_{b}-\tau )\mathrm{sinc}(2(W-f_{b,2})\tau )(1-\frac{\tau }{\left|T_{b}\right|}) \end{array}$$

When two LFM-FT signals with distinct CIDs $\left(b,b^{\prime }\right)$ are synchronised in time, its cross-correlation function can be expressed as follows:

(4) $$\begin{array}{cc} \rho_{b,b^{\prime }}^{\mathrm{LFM}\_\mathrm{FT}}= & \frac{1}{T_{b}}\int_{0}^{\frac{T_{b}}{2}}x_{b,1}^{\mathrm{LFM}\_\mathrm{FT}}(s)x_{b^{\prime },1}^{\mathrm{LFM}\_\mathrm{FT}}{\left(s\right)}^{*}ds+\frac{1}{T_{b}}\int_{\frac{T_{b}}{2}}^{T_{b}}x_{b,1}^{\mathrm{LFM}\_\mathrm{FT}}(s)x_{b^{\prime },2}^{\mathrm{LFM}\_\mathrm{FT}}{\left(s\right)}^{*}ds\\ = & \rho_{b,b^{\prime },1}^{\mathrm{LFM}\_\mathrm{FT}}+\rho_{b,b{,}^{\prime }2}^{\mathrm{LFM}\_\mathrm{FT}} \end{array}$$

The cross-correlation function for the first half of the preamble is expressed as

(5) $$\begin{array}{cc} \rho_{b,b^{\prime },1}^{\mathrm{LFM}\_\mathrm{FT}}= & \frac{1}{T_{b}}\int_{0}^{\frac{T_{b}}{2}}x_{b,1}^{\mathrm{LFM}\_\mathrm{FT}}(s)x_{b^{\prime },1}^{\mathrm{LFM}\_\mathrm{FT}}{\left(s\right)}^{*}ds\\ = & \frac{1}{T_{b}}\int_{0}^{\frac{T_{b}}{2}}e^{j\pi (2f_{b,1}s+\frac{\beta_{b,1}}{T_{b}/2}s^{2})}e^{j\pi {\left(2f_{b^{\prime },1}s+\frac{\beta_{b^{\prime },1}}{T_{b}/2}s^{2}\right)}^{*}}ds\\ = & \frac{1}{T_{b}}\int_{0}^{\frac{T_{b}}{2}}e^{j\pi (\mu_{b,1}s^{2})}ds \end{array}$$

where $\mu_{b,1}=2\Delta B_{b_{1},{b^{\prime }}_{1}}/T_{b}$. Here, $\Delta B_{b_{1},{b^{\prime }}_{1}}=\beta_{b^{\prime },1}-\beta_{b,1}$ and $\beta_{b^{\prime },1}\geq \beta_{b,1}$. In (5), the starting frequencies of two preambles, $f_{b,1}$ and $f_{b^{\prime },1}$, are set to zero. With a change in variable $v=\sqrt{2\mu_{b,1}}s$, (5) can be expressed as

(6) $$\rho_{b,b^{\prime },1}^{\mathrm{LFM}\_\mathrm{FT}}=\frac{1}{2\sqrt{T_{b}\Delta B_{b_{1},{b^{\prime }}_{1}}}}\int_{0}^{\sqrt{T_{b}\Delta B_{b_{1},{b^{\prime }}_{1}}}}\cos(\frac{\pi v^{2}}{2})dv\;\Rightarrow \;\frac{1}{2\sqrt{T_{b}\Delta B_{b_{1},{b^{\prime }}_{1}}}}C(\sqrt{T_{b}\Delta B_{b_{1},{b^{\prime }}_{1}}})$$

where $C(x)=\int_{0}^{x}\cos(\pi v^{2}/2)dv$. Here, C(x) represents the Fresnel’s cosine integral [23]. The cross-correlation function for the second half of the preamble is given as

(7) $$\begin{array}{cc} \rho_{b,b^{\prime },2}^{\mathrm{LFM}\_\mathrm{FT}}= & \frac{1}{2\sqrt{T_{b}\Delta B_{b_{2},{b^{\prime }}_{2}}}}\{\cos(\pi \frac{T_{b}\Delta B_{b_{2},{b^{\prime }}_{2}}}{2})C(\sqrt{T_{b}\Delta B_{b_{2},{b^{\prime }}_{2}}})\}+\\ & \frac{1}{2\sqrt{T_{b}\Delta B_{b_{2},{b^{\prime }}_{2}}}}\{\sin(\pi \frac{T_{b}\Delta B_{b_{2},{b^{\prime }}_{2}}}{2})S(\sqrt{T_{b}\Delta B_{b_{2},{b^{\prime }}_{2}}})\} \end{array}$$

where $S(x)=\int_{0}^{x}\sin(\pi v^{2}/2)dv$. Here, S(x) represents the Fresnel’s sine integral [23]. In addition, $\Delta B_{b_{2},{b^{\prime }}_{2}}=\beta_{b^{\prime },2}-\beta_{b,2}$ and $\beta_{b^{\prime },2}\geq \beta_{b,2}$. Note that $\Delta B_{b_{1},{b^{\prime }}_{1}}=\Delta B_{b_{2},{b^{\prime }}_{2}}=\Delta B$ because $\Delta B_{b_{1},{b^{\prime }}_{1}}$ is always equal to $\Delta B_{b_{2},{b^{\prime }}_{2}}$. Combining (6) and (7), the cross-correlation of LFM-FT signals -CST is given as

(8) $$\rho_{b,b^{\prime }}^{\mathrm{LFM}\_\mathrm{FT}}=\frac{1}{2\sqrt{T_{b}\Delta B}}\{C(\sqrt{T_{b}\Delta B})(1+\cos(\pi \frac{T_{b}\Delta B}{2}))+\sin(\pi \frac{T_{b}\Delta B}{2})S(\sqrt{T_{b}\Delta B})\}$$

**Figure 2:**
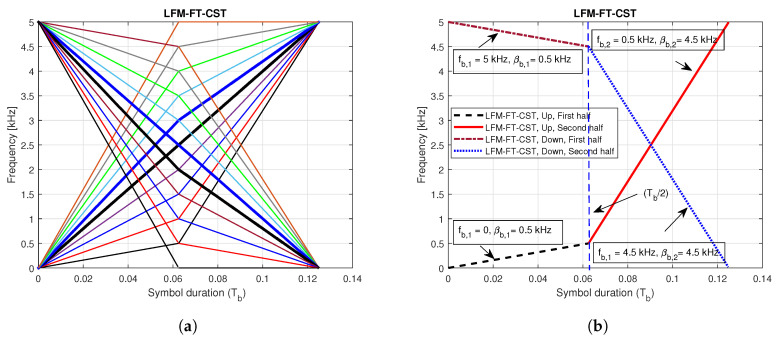
Instantaneous frequencies of LFM-FT signals for multiple cell IDs (CIDs). (**a**) Multiple CIDs; (**b**) Two CIDs.

By constructing the preamble with two different frequency sweeping parameters and starting frequencies, we can assign full bandwidth to UBSs, which improves detection probability and correct timing estimation. Figure 2a shows the instantaneous frequencies of LFM-FT signals with different CIDs. Figure 2b illustrates the case with two CIDs. In this figure, the first signal, characterised by positive slopes, has the parameters ($f_{b,1}$ = 0 kHz, $\beta_{b,1}$ = 0.5 kHz) for the first half and ($f_{b,2}$ = 0.5 kHz, $\beta_{b,2}$ = 4.5 kHz) for the second half. The second signal, characterised by negative slopes, has the parameter ($f_{b,1}$ = 5 kHz, $\beta_{b,1}$ = 0.5 kHz) for the first half and ($f_{b,2}$ = 4.5 kHz, $\beta_{b,2}$ = 4.5 kHz) for the second half.

**Figure 3:**
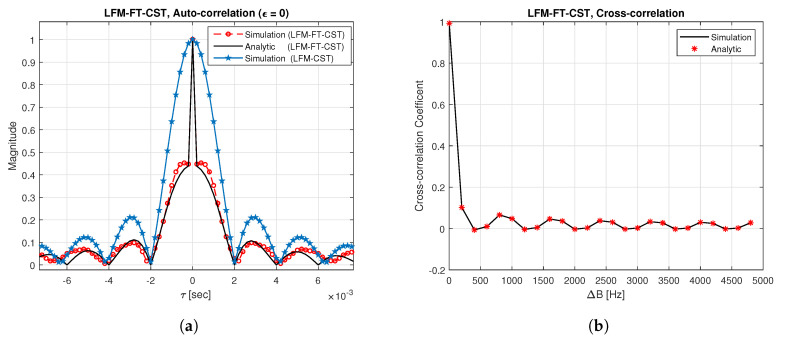
Auto-correlation and cross-correlation of LFM-FT signals. (**a**) Auto-correlation; (**b**) Cross-correlation.

Figure 3a displays the auto-correlation plot of LFM-FT signals when the first signal with positive slopes in Figure 2b is used. The analytic result is obtained by (3). From this figure, it can be seen that the simulation result exhibits good agreement with the analytic result. In this figure, the result is also compared with the previous LFM-CST ($\beta_{b}$ = 0.5 kHz). The LFM-FT-CST produces a sharper peak than the LFM-CST because a small bandwidth is used in the LFM-CST. Note that a different bandwidth is assigned to the UBSs in the LFM-CST, whereas full bandwidth is always assigned to all the UBSs in the LFM-FT-CST. A more accurate timing estimate is obtained as the peak in the auto-correlation function becomes sharper. Figure 3b depicts the cross-correlation plot of LFM-FT-CST when two LFM-FT signals with distinct CIDs $\left(b,b^{\prime }\right)$ are used. The analytic result is obtained by (8). From this figure, it can be seen that the simulation result agrees well with the analytic result. It can be also seen that the cross-correlation level is maximum when the separation (ΔB) between the two signals is zero. As ΔB increases, the cross-correlation level decreases, resulting in the reduction of interferences from other UBSs. The cross-correlation level is less than 10% if ΔB is greater than 250 Hz. Thus, as ΔB increases, the CID detection probability will increase. However, the number of possible CIDs decreases with an increase in ΔB.

**Figure 4:**
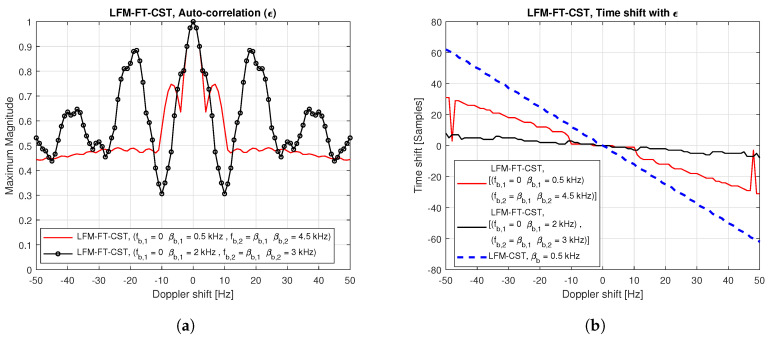
Auto-correlation of LFM-FT signal in the presence of Doppler shift: maximum magnitude and time shift. (**a**) Maximum magnitude vs. Doppler shift; (**b**) Time shift vs. Doppler shift.

Figure 4 exhibits the auto-correlation plot of LFM-FT signals when the Doppler shift exists. In this figure, only the simulation results are shown because analytic solution cannot be derived for the LFM-FT-CST in the presence of Doppler shift. Figure 4a,b indicate the maximum magnitude and time shift when different starting frequencies $\left(f_{b,1},f_{b,2}\right)$ and sweeping parameters $\left(\beta_{b,1},\beta_{b,2}\right)$ are used for multiple CIDs. From these figures, it can be seen that, when ϵ = 0, the maximum magnitude is 1, regardless of parameter values, and time shift does not occur. As ϵ increases, the maximum magnitude fluctuates and time shift increases. For example, the time shift is one sample at ϵ = 10 Hz when $(f_{b,1}=0,\beta_{b,1}$ = 0.5 kHz) and $(f_{b,2}=\beta_{b,1},\beta_{b,2}$ = 4.5 kHz) are used. However, the amount of time shift occurring in the LFM-FT-CST is small compared to that in the LFM-CST ($\beta_{b}$ = 0.5 kHz), as illustrated in Figure 4b.

### 3.2. Cell Search Technique Based on LFM in Frequency Domain (LFM-FF)

The LFM-FF-CST is proposed to increase CID detection probability and accurate timing in the presence of Doppler shift. In the proposed LFM-FF-CST, multiple LFM-FF signals are generated in the frequency domain with different frequency sweeping parameters. The LFM-FF signal is sampled and mapped to subcarriers in the frequency-domain resource grid. It is shown that its time-domain signal obtained by IDFT has an ideal AF and CAF in the presence of Doppler shift when the parameters are selected appropriately. Although the LFM-FF signal is generated and mapped to subcarriers in the frequency domain, we start with the time-domain description because of its familiarity. A time-domain LFM waveform with a symbol duration, $T_{b}$, for the *b*th UBS is given by [21]

(9) $$\begin{array}{cc} x_{b}^{\mathrm{LFM}\_\mathrm{FF}}(t)=e^{j\pi \frac{\beta_{b}}{T_{b}}t^{2}}, & 0\leq t<T_{b} \end{array}$$

where $-W\leq \beta_{b}\leq W$ denotes the frequency sweeping parameter. The LFM-FF signal is generated by sampling the LFM waveform and mapping the samples in the frequency domain. Let $T_{s}=T_{b}/M_{s}$ be the sampling interval. Here, $M_{s}$ denotes the number of samples. Subsequently, the sampled version of (9) is given by

(10) $$x_{b}^{\mathrm{LFM}\_\mathrm{FF}}(k)=\{\begin{array}{cc} e^{\frac{j\pi }{M_{s}}\beta_{b}T_{s}k^{2}}, & 0\leq k\leq M_{s}-1\\ 0, & \mathrm{elsewhere} \end{array}$$

The sampled signal in the frequency domain is transformed into the time-domain signal via an IDFT as follows:

(11) $$\begin{array}{cc} {\tilde{x}}_{b}(n)= & \frac{1}{M_{s}}\sum_{k=0}^{M_{s}-1}e^{\frac{j\pi }{M_{s}}\kappa_{b}k^{2}}e^{\frac{j2\pi nk}{M_{s}}}\\ = & \frac{1}{M_{s}}\sum_{k=0}^{M_{s}-1}e^{\frac{j\pi }{M_{s}}\kappa_{b}[{\left(k+n\kappa_{b}^{-1}\right)}^{2}-{\left(n\kappa_{b}^{-1}\right)}^{2}]} \end{array}$$

where $\kappa_{b}=\beta_{b}T_{s}$. Assuming a high sampling rate, (11) becomes

(12) $${\tilde{x}}_{b}(n)=\frac{1}{M_{s}}e^{-\frac{j\pi }{\kappa_{b}M_{s}}n^{2}}\int_{0}^{M_{s}-1}e^{\frac{j\pi }{M_{s}}\kappa_{b}{\left(k+n\kappa_{b}^{-1}\right)}^{2}}dk$$

Equation (12) can be rewritten as

(13) $${\tilde{x}}_{b}(n)=\frac{1}{M_{s}}e^{-\frac{j\pi }{\kappa_{b}M_{s}}n^{2}}\int_{n\kappa_{b}^{-1}}^{M_{s}+n\kappa_{b}^{-1}-1}e^{\frac{j\pi }{M_{s}}\kappa_{b}\eta^{2}}d\eta$$

where $\eta =k+n\kappa_{b}^{-1}$. Equation (13) can be expressed as

(14) $${\tilde{x}}_{b}(n)=\frac{1}{M_{s}}e^{-\frac{j\pi }{\kappa_{b}M_{s}}n^{2}}\int_{n\sqrt{\frac{j\pi }{\kappa_{b}M_{s}}}}^{(M_{s}+n\kappa_{b}^{-1}-1)\sqrt{\frac{j\pi \kappa_{b}}{M_{s}}}}e^{u^{2}}\sqrt{\frac{M_{s}}{j\pi \kappa_{b}}}du$$

where $u=\eta \sqrt{\frac{j\pi \kappa_{b}}{M_{s}}}$. Using the Fresnel integral, (14) can be expressed as

(15) $$\begin{array}{cc} {\tilde{x}}_{b}(n)= & \frac{1}{2\sqrt{j\kappa_{b}M_{s}}}e^{-\frac{j\pi }{\kappa_{b}M_{s}}n^{2}}[erfi((M_{s}+\frac{n}{\kappa_{b}}-1)\sqrt{\frac{j\pi \kappa_{b}}{M_{s}}})-erfi(n\sqrt{\frac{j\pi }{\kappa_{b}M_{s}}})]\\ = & \frac{1}{2\sqrt{j\kappa_{b}M_{s}}}e^{-\frac{j\pi }{\kappa_{b}M_{s}}n^{2}}(1-j)[C(\frac{(1+j)z1}{\sqrt{\pi }})-jS(\frac{(1+j)z1}{\sqrt{\pi }})-C(\frac{(1+j)z2}{\sqrt{\pi }})+jS(\frac{(1+j)z2}{\sqrt{\pi }})]\\ = & \frac{1}{2\sqrt{j\kappa_{b}M_{s}}}e^{-\frac{j\pi }{\kappa_{b}M_{s}}n^{2}}(1-j)[Z(\frac{(1+j)z1}{\sqrt{\pi }})-Z(\frac{(1+j)z2}{\sqrt{\pi }})] \end{array}$$

where $z1=(M_{s}+\frac{n}{\kappa_{b}}-1)\sqrt{\frac{j\pi \kappa_{b}}{M_{s}}}$ and $z2=k\sqrt{\frac{j\pi }{\kappa_{b}M_{s}}}$. Here, Z represents the complex conjugate Fresnel integral [24]. To analyse auto-correlation property of the LFM-FF signal in the presence of a Doppler shift, the AF of the LFM-FF signal is derived in the discrete-time domain as follows:

(16) $$\begin{array}{cc} \rho_{b}^{\mathrm{LFM}\_\mathrm{FF}}(n,\varepsilon )= & \sum_{m=0}^{M_{s}-1}{\tilde{x}}_{b}(m){\tilde{x}}_{b}{\left(m-n\right)}^{*}e^{\frac{2j\pi \varepsilon m}{M_{s}}}\\ = & \frac{1}{M_{s}^{2}}\sum_{m=0}^{M_{s}-1}e^{-\frac{j\pi }{\kappa_{b}M_{s}}(m^{2}-{\left(m-n\right)}^{2})}e^{\frac{j\pi \varepsilon m}{M_{s}}}\sum_{d=0}^{M_{s}-1}\sum_{g=0}^{M_{s}-1}e^{\frac{j\pi \kappa_{b}}{M_{s}}{\left(d+\frac{m}{\kappa_{b}}\right)}^{2}}e^{-\frac{j\pi \kappa_{b}}{M_{s}}{\left(d+\frac{\left(m-n\right)}{\kappa_{b}}\right)}^{2}}\\ = & \frac{1}{M_{s}^{2}}e^{\frac{j\pi n^{2}}{\kappa_{b}M_{s}}}\sum_{m=0}^{M_{s}-1}e^{-\frac{j2\pi }{M_{s}}(\frac{n}{\kappa_{b}}-\varepsilon )}\sum_{d=0}^{M_{s}-1}\sum_{g=0}^{M_{s}-1}e^{\frac{j\pi \kappa_{b}}{M_{s}}[d^{2}-{\left(g-\frac{n}{\kappa_{b}}\right)}^{2}+\frac{2}{\kappa_{b}}(d-g+\frac{n}{\kappa_{b}})m]}\\ = & \frac{1}{M_{s}^{2}}e^{\frac{j\pi n^{2}}{\kappa_{b}M_{s}}}\sum_{d=0}^{M_{s}-1}\sum_{g=0}^{M_{s}-1}\sum_{m=0}^{M_{s}-1}e^{\frac{j\pi \kappa_{b}d^{2}}{M_{s}}}e^{\frac{-j\pi \kappa_{b}}{M_{s}}{\left(g-\frac{n}{\kappa_{b}}\right)}^{2}}e^{\frac{j2\pi }{M_{s}}(\varepsilon +d-g)m} \end{array}$$

Equation (16) can be expressed as

(17) $$\rho_{b}^{\mathrm{LFM}\_\mathrm{FF}}(n,\varepsilon )=\frac{1}{M_{s}^{2}}e^{\frac{j\pi n^{2}}{\kappa_{b}M_{s}}}\{\begin{array}{cc} \sum_{d=0}^{M_{s}+n-1}\sum_{g=0}^{M_{s}+n-1}e^{\frac{j\pi \kappa_{b}d^{2}}{M_{s}}}e^{\frac{-j\pi \kappa_{b}}{M_{s}}{\left(g-\frac{n}{\kappa_{b}}\right)}^{2}}\frac{1-e^{j2\pi \varepsilon }}{1-e^{\frac{j2\pi }{M_{s}}(\varepsilon +d-g)}} & -M_{s}<n\leq 0\\ \sum_{d=0}^{M_{s}-n-1}\sum_{g=0}^{M_{s}-n-1}e^{\frac{j\pi \kappa_{b}d^{2}}{M_{s}}}e^{\frac{-j\pi \kappa_{b}}{M_{s}}{\left(g-\frac{n}{\kappa_{b}}\right)}^{2}}\frac{1-e^{j2\pi \varepsilon }}{1-e^{\frac{j2\pi }{M_{s}}(\varepsilon +d-g)}} & 0<n<M_{s} \end{array}$$

From (17), it can be seen that the magnitude of the AF of the LFM-FF signal varies with respect to $\beta_{b}$ (or $\kappa_{b})$ and ϵ. When d=g and n=0, (17) can be approximated as

(18) $$\rho_{b}^{\mathrm{LFM}\_\mathrm{FF}}(0,\varepsilon )\approx \frac{\mathrm{sinc}(\pi \varepsilon )}{\mathrm{sinc}(\frac{\pi \varepsilon }{M_{s}})}$$

From (18), it can be seen that the magnitude is 1 at the origin (n=0,ϵ=0) The result in (18) is only true when $\kappa_{b}=1\;(\beta_{b}=W)$ For a small value of Doppler shift (ϵ), the magnitude of AF with no timing error can be approximated as $\left|\rho_{b}^{\mathrm{LFM}\_\mathrm{FF}}(0,\varepsilon )|\approx |\mathrm{sinc}(\varepsilon )\right|$, which is a sinc function of ϵ. Next, to analyse the cross-correlation property of two LFM-FF signals with distinct CIDs $\left(b,b^{\prime }\right)$ in the presence of a Doppler shift, the CAF of the LFM-FF signal is derived in the discrete-time domain as follows:

(19) $$\begin{array}{c} \rho_{b,b^{\prime }}^{\mathrm{LFM}\_\mathrm{FF}}(n,\varepsilon )=\sum_{m=0}^{M_{s}-1}{\tilde{x}}_{b}(m){\tilde{x}}_{b^{\prime }}{\left(m-n\right)}^{*}e^{\frac{2j\pi \varepsilon m}{M_{s}}}\\ =\frac{1}{M_{s}^{2}}e^{\frac{j\pi n^{2}}{{\kappa_{b}}^{\prime }M_{s}}}\;\{\begin{array}{cc} \sum_{d=0}^{M_{s}+n-1}\sum_{g=0}^{M_{s}-1}e^{\frac{j\pi {\kappa_{b}}^{\prime }d^{2}}{M_{s}}}e^{-\frac{j\pi {\kappa_{b}}^{\prime }}{M_{s}}{\left(g-\frac{n}{{\kappa_{b}}^{\prime }}\right)}^{2}}\frac{1-e^{j2\pi \varepsilon }}{1-e^{\frac{j2\pi }{M_{s}}(\varepsilon +d-g)}} & -M_{s}<n\leq 0\\ \sum_{d=0}^{M_{s}-1}\sum_{g=0}^{M_{s}-1}e^{\frac{j\pi {\kappa_{b}}^{\prime }d^{2}}{M_{s}}}e^{-\frac{j\pi {\kappa_{b}}^{\prime }}{M_{s}}{\left(g-\frac{n}{{\kappa_{b}}^{\prime }}\right)}^{2}}\frac{1-e^{j2\pi \varepsilon }}{1-e^{\frac{j2\pi }{M_{s}}(\varepsilon +d-g)}} & 0<n<M_{s} \end{array} \end{array}$$

where ${\kappa_{b}}^{\prime }=\beta_{b}^{\prime }T_{s}$, $\varepsilon =f_{D}T_{b}$, and $d,g\in [0,M_{s}-1]$. Here, $f_{D}$ denotes Doppler shift. The separation $\Delta \kappa_{b}({\kappa_{b}}^{\prime }-\kappa_{b})$ between the two LFM-FF signals with $\beta_{b}$ and $\beta_{b}^{\prime }$ must be cautiously selected to obtain a low correlation between them while supporting a large number of preambles. The upper bound of CAF can be derived as follows:

(20) $$\xi (\Delta \kappa_{b})=2\sqrt{\frac{C_{\max}^{2}+S_{\max}^{2}}{\pi \Delta \kappa_{b}M_{s}}}$$

where $C_{\max}$ and $S_{\max}$ denote the maximum values of cosine and sine Fresnel integrals, and are given as 0.9775 and 0.894, respectively. As can be seen from (20), the upper bound is independent of the Doppler shift, indicating that the Doppler shift does not significantly influence the magnitude of CAF. However, the values of $T_{b}$ and $\Delta \kappa_{b}$ have a significant effect on the magnitude of CAF. The larger the $\Delta \kappa_{b}M_{s}$, the smaller the upper bound. Thus, as the separation $\Delta \kappa_{b}$ between the two LFM-FF signals increases, the cross-correlation level decreases, resulting in the reduction of interferences from other UBSs. However, the number of possible CIDs decreases with an increase in $\Delta \kappa_{b}$.

**Figure 5:**
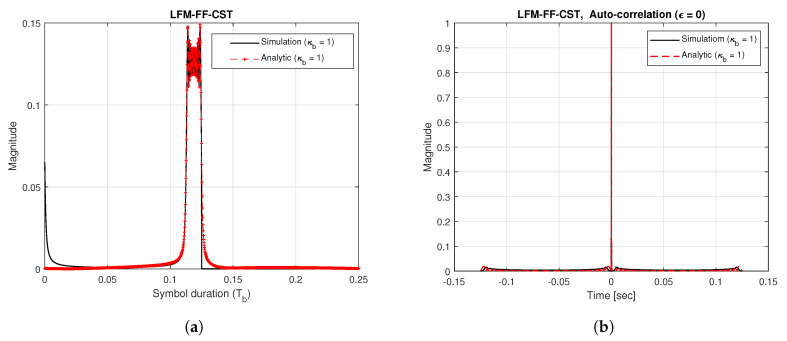
Time-domain waveform and auto-correlation of LFM-FF signal. (**a**) Time-domain waveform; (**b**) Auto-correlation.

Figure 5a shows the time-domain waveform of the LFM-FF signal generated using $\Delta \kappa_{b}$ = 1. This figure also shows a comparison of the analytical solution obtained in (15) with the simulation results. The results show that the analytical solution agrees well with the simulation results. Figure 5b shows the auto-correlation function of the LFM-FF signal when the Doppler shift does not exist. From this figure, it can be seen that the analytical solution obtained using (17) with (ϵ=0) agrees well with the simulation results. It can also be seen that the LFM-FF signal produces a sharper peak than the LFM-FT signal in Figure 3a, resulting in higher accuracy in timing estimation.

**Figure 6:**
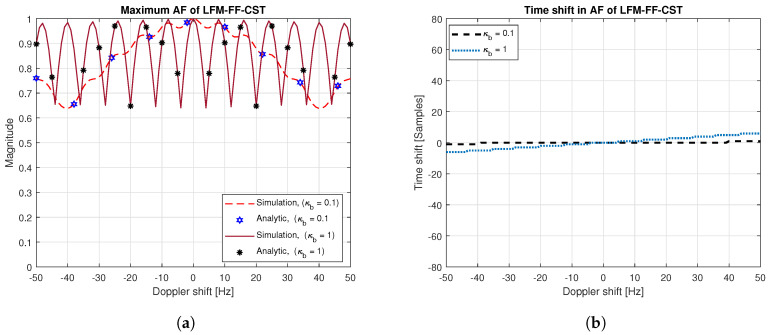
AF of LFM-FF signal: maximum magnitude and time shift. (**a**) Maximum magnitude vs. Doppler shift; (**b**) Time shift vs. Doppler shift.

Figure 6a,b show the maximum magnitude and time shift of AF of the LFM-FF signal when the Doppler shift exists. Here, the value of $\Delta \kappa_{b}$ is set to {0.1,1}, corresponding $\beta_{b}$ is {0.5,5} kHz, respectively. From Figure 6a, it can be seen that the analytical solution given in (17) agrees well with simulation result for all values of Doppler shift. When the Doppler shift is zero, the maximum magnitude is 1 and time shift does not occur. As the Doppler shift increases, the maximum magnitude fluctuates and the time shift increases. Furthermore, as $\Delta \kappa_{b}$ increases, the variance of the maximum magnitude in Figure 6a increases and the time shift in Figure 6b increases. For example, when $\Delta \kappa_{b}$ is {0.1,1}, the time shift becomes {1,5} samples for a Doppler shift of 41 Hz. However, the variance of maximum magnitude and time shift in the LFM-FF-CST are smaller than those in the LFM-FT-CST or LFM-CST (Figure 4).

**Figure 7:**
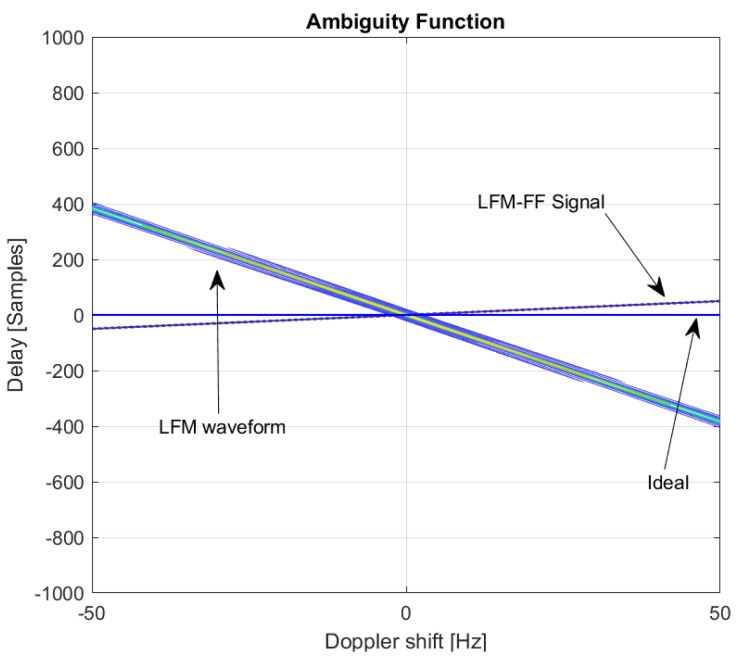
Contour plot of AFs: LFM, LFM-FF, Ideal.

Figure 7 shows a comparison of the contour plots of AFs of the LFM and LFM-FF signals. As can be seen in the figure, the AF of the LFM waveform is a skewed version of a triangular ridge in the delay-Doppler plane, implying that the target is well detected in the presence of a Doppler shift. The LFM waveform is widely used in surveillance applications because the target is more likely to be detected in high Doppler environments. However, when there is a Doppler mismatch, its peak will not occur at the correct time. The peak will be shifted by the amount of samples proportional to the Doppler shift. On the other hand, the ideal AF for target detection with correct timing will be “a line” located on the horizontal (Doppler shift) axis, as shown in Figure 7. In this case, the AF will produce a high peak at the correct time, regardless of the amount of Doppler shift. From this figure, it can be seen that the proposed LFM-FF signal possesses the characteristics of the Ideal AF, producing a high peak at the correct timing in the presence of a Doppler shift.

**Figure 8:**
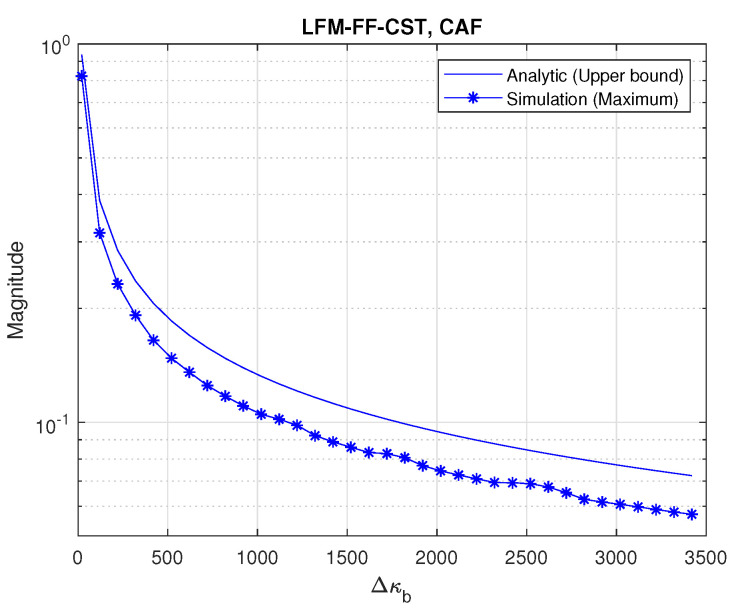
Cross-ambiguity function (CAF) of LFM-FF signals vs. $\Delta \kappa_{b}$.

Figure 8 depicts the CAF property of two LFM-FF signals with distinct CIDs $\left(b,b^{\prime }\right)$ when $\Delta \kappa_{b}$ varies. Here, $T_{b}$ is set to 0.125 s. The upper bound of CAF, obtained by (20), is slightly higher than the maximum level of cross-correlation, obtained by the simulation. The cross-correlation level is maximum when $\Delta \kappa_{b}$ is zero. As $\Delta \kappa_{b}$ increases, the cross-correlation level decreases, resulting in an increase in CID detection probability. Thus, in the LFM-FF-CST, there is a trade-off between the cross-correlation level and the number of possible CIDs, during the selection of the value of $\Delta \kappa_{b}$.

## 4. Simulations

In this section, the performance of two proposed CSTs (LFM-FT and LFM-FF) for UAC systems is evaluated through a computer simulation. For the simulation, a three-cell model is used with a cell radius $R_{c}$ equals to 5 km and frequency reuse factor of 1 [22]. Here, the UE is located at the edge of cell 1, and the distance between UE and UBS1/UBS2/UBS3 is 4.5/5.14/5.39 km. With this configuration, UE needs to detect and select UBS 1 as a serving cell with UBS2 and UBS3 as interfering cells. In addition, as large discrepancy in time delay of arrival exists due to the low speed of the acoustic wave, the simulation model is assumed to be an asynchronous system. For OFDM parameters, channel bandwidth, subcarrier spacing, and carrier frequency $\left(f_{c}\right)$ are set to 5 kHz (from 22 kHz to 27 kHz), 9.76 Hz, and 24.5 kHz, respectively. The OFDM symbol duration including CP is set to 0.125 s. The symbol durations of LFM-FT and LFM-FF preambles are also set to 0.125 s. For the LFM-FT-CST, the following three parameter sets are assigned to three UBSs: ($f_{b,1}=0$, $\beta_{b,1}$ = 1.5 kHz, $f_{b,2}$ = 1.5 kHz, $\beta_{b,2}$ = 3.5 kHz) ($f_{b,1}=0$, $\beta_{b,1}$ = 1 kHz, $f_{b,2}$ = 1 kHz, $\beta_{b,2}$ = 4 kHz) and ($f_{b,1}=0$, $\beta_{b,1}$ = 0.5 kHz, $f_{b,2}$ = 0.5 kHz, $\beta_{b,2}$ = 4.5 kHz). For the LFM-FF-CST, the value of $\beta_{b}$ is set to 0.5 kHz for UBS1, 1 kHz for UBS2, and 1.5 kHz for UBS3.

Large-scale fading and small-scale fading are considered for underwater channel model. The large-scale fading is characterized by the path loss which is dependent on distance and frequency. The path loss of underwater acoustic wave at a distance *x* from the transmitter is given by [25].

(21) $$PL_{\mathrm{dB}}=10\gamma \log_{10}(\frac{x}{x_{o}})+(x-x_{o})a_{\mathrm{dB}/\mathrm{km}}\left(f_{c}\right)$$

where γ, $x_{o}$, and $a_{\mathrm{dB}/\mathrm{km}}\left(f_{c}\right)$ denote spreading loss, reference distance, and absorption coefficient given by Throp’s formula, respectively. The absorption coefficient is given as

(22) $$a_{\mathrm{dB}/\mathrm{km}}\left(f_{c}\right)=\frac{0.11f_{c}^{2}}{1+f_{c}}+\frac{44f_{c}^{2}}{4100+f_{c}^{2}}+2.75\times 10^{-4}f_{c}^{2}+0.003$$

The first and second terms in (21) represent the spreading loss and absorption loss, respectively. The spreading loss consists of cylindrical spreading loss in shallow water and spherical spreading loss in deep water. The range of γ is between 1 and 2. For a practical system, γ = 1.5 [25]. The small-scale fading is characterized by the time-varying and sparse multipath owing to the low speed of acoustic wave and propagation in multiple trajectories.

The performance of the proposed CSTs is evaluated under AWGN and multiple channel models. In this study, the Bellhop channel simulator widely used in underwater acoustic communication is used to simulate the multipath channels [26,27]. The values of the parameters provided in the field experimental site (distance: 2 km, depth: 50 m) are used to setup the Bellhop channel simulator. The generated multipath channel consists of 12 paths, each experiencing Rician fading with different *K* factors. Table 1 lists the corresponding path delay and magnitude, and *K* factors generated by the simulator. Further, a Doppler shift is applied to both the LOS $\left(f_{dLOS}\right)$ and NLOS $\left(f_{dNLOS}\right)$ components, which makes the channel time-varying.

**Figure 9:**
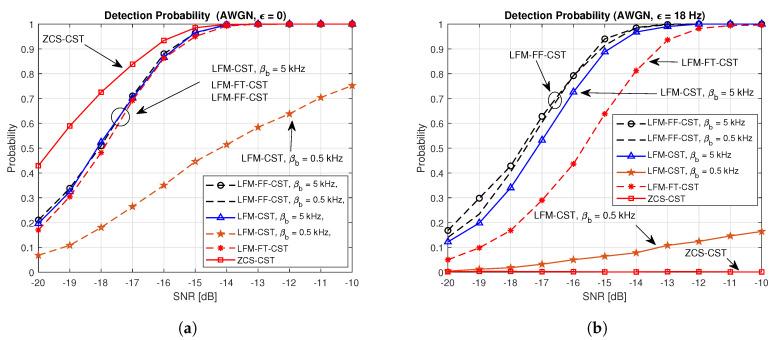
Detection probabilities of proposed and conventional techniques in AWGN channel. (**a**) AWGN, ε=0Hz; (**b**) AWGN, ε=18Hz.

Figure 9 compares the detection probabilities of the proposed techniques (LFM-FT-CST and LFM-FF-CST) and conventional techniques (ZCS-CST and LFM-CST) for a AWGN channel. Figure 9a,b illustrates the cases of ϵ=0 and ϵ=18 Hz, respectively. In the ZCS-CST, cell searching is performed separately from preamble detection. Cell searching is performed using the received ZCS with the timing estimated by the LFM preamble. In the LFM-CST, LFM-FT-CST, and LFM-FF-CST, the preamble (timing) detection and CID detection are performed jointly using parallel correlators. From Figure 9a, it can be seen that the performance of the LFM-CST varies depending on the parameter assigned to the UBS. The probability of correctly detecting the CID of serving cell (UBS1) is high when a full bandwidth is assigned to the frequency sweeping parameter $\left(\beta_{b}=5\right)$ kHz. However, the detection probability decreases significantly for a small value of the frequency sweeping parameter $\left(\beta_{b}=0.5\right)$ kHz. In a practical situation, full bandwidth can be assigned to only one UBS. It can be seen from Figure 9a that the performances of LFM-CST $\left(\beta_{b}=5\right)$ kHz, LFM-FT-CST ($f_{b,1}=0$, $\beta_{b,1}$ = 1.5 kHz, $f_{b,2}$ = 1.5 kHz, $\beta_{b,2}$ = 3.5 kHz), and LFM-FF-CST $(\beta_{b}$ = 0.5 or 5 kHz) are similar. The best performance is obtained when the ZCS-CST is used because the ZCS has an ideal auto-correlation property in the absence of a Doppler shift. However, as can be seen in Figure 9b, the performance of the ZCS-CST degrades significantly, resulting in failure to detect CID, when a Doppler shift exists. Here, a Doppler shift of 18 Hz is considered because the average speed of AUVs is 1 m/s [28]. The speed of 1 m/s corresponds to a Doppler shift of 18 Hz in an underwater environment. The LFM-CST with a small value of the frequency sweeping parameter $(\beta_{b}$ = 0.5 kHz) also exhibits poor performance, less than 20% detection probability at an SNR of −10 dB. The LFM-FF-CST exhibits the best performance, regardless of its parameter value $(\beta_{b}$ = 0.5 or 5 kHz). The LFM-FT-CST CST exhibits a slightly poorer performance than the LFM-FF-CST because it is more sensitive to the Doppler shift than the LFM-FF-CST, as can be seen from Figure 4 and Figure 6.

**Figure 10:**
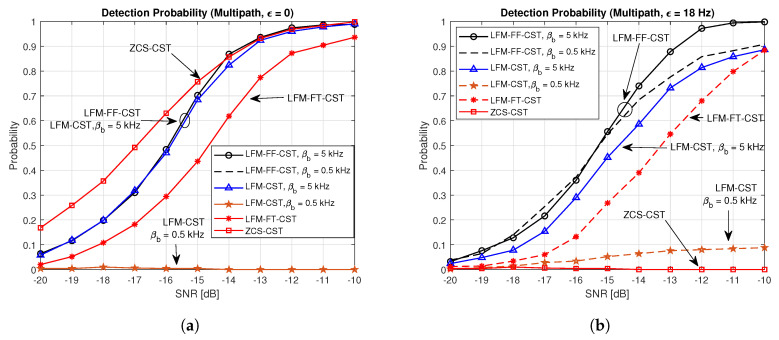
Detection probabilities of proposed and conventional techniques in multipath channel. (**a**) Multipath channel, ε=0Hz; (**b**) Multipath channel, ε=18Hz.

Figure 10 shows a comparison of the detection probabilities of the proposed techniques and conventional techniques in the multipath channels generated by the Bellhop channel simulator. Figure 10a,b depict the cases of ϵ=0 and ϵ=18 Hz, respectively. The performance in Figure 10a is similar to the one in Figure 9a. The performance of the LFM-CST varies depending on its parameter. The detection probability is almost zero when $\beta_{b}$ = 0.5 kHz and is high when $\beta_{b}$ = 5 kHz. The performances of the LFM-CST $(\beta_{b}$ = 5 KHz) and LFM-FF-CST are similar. The LFM-FT-CST ($f_{b,1}=0$, $\beta_{b,1}$ = 1.5 kHz, $f_{b,2}$ = 1.5 kHz, $\beta_{b,2}$ = 3.5 kHz) exhibits a slightly poorer performance than these. The best performance is obtained when the ZCS-CST is used. However, when a Doppler shift exists, the performance of the ZCS-CST degrades significantly, resulting in failure to detect CID, as can be seen in Figure 10b. When a Doppler shift exists, the detection probability of LFM-CST with $\beta_{b}$ = 0.5 kHz is less than 10% at an SNR of −10 dB. The LFM-FF-CST can achieve the best performance (above 90% detection probability at the SNR of −10 dB), regardless of its parameter value $(\beta_{b}$ = 0.5 or 5 KHz). The LFM-CST $(\beta_{b}$ = 5 kHz) and LFM-FT-CST exhibits a slightly poorer performance than the LFM-FF-CST.

## 5. Conclusions

In this paper, two different types of cell search techniques (LFM-FT-CST and LFM-FF-CST) for UAC systems were proposed to detect the CID of UBS and estimate correct timing in the presence of a Doppler shift. The LFM-FT-CST was proposed by modifying the previous LFM-CST in the time domain so that full bandwidth can be used in the design of preambles with different frequency sweeping parameters. The auto-correlation and cross-correlation functions of LFM-FT-CST were derived and shown to exhibit good agreement with the simulation results. The LFM-FF-CST was generated by mapping the sampled LFM waveform to subcarriers in the frequency domain resource grid. The AF and CAF of the LFM-FF-CST were derived to examine its auto-correlation and cross-correlation properties in the existence of a Doppler shift, and they showed good agreement with the simulation results. In addition, the upper bound of CAF was analysed to determine the cross-correlation (interference) level and number of possible sequences. It was revealed that there is a trade-off between the number of preambles and interference level with the selection of Δβ. It was also highlighted that the LFM-FF-CST produces high peaks at the correct timing in the existence of a Doppler shift, which is an ideal characteristic of AF. It was confirmed by simulation that the proposed technique performs better than the previous techniques in both AWGN and multipath channels when a Doppler shift exists. It was shown that the LFM-FF-CST achieves the best performance (above 90% detection probability at an SNR of −10 dB) in the presence of a Doppler shift, regardless of its parameter value. 

## Figures and Tables

**Table 1:** Multipath channel generated by Bellhop channel model.

Path Delay [ms]	Magnitude	*K* Factor [dB]
0	0.537	43.50
0.06	0.580	52.48
1.50	0.427	19.95
1.56	0.267	119.31
1.67	0.274	61.68
6.33	0.183	12.59
6.39	0.073	79.43
6.61	0.0650	31.62
6.68	0.0303	50.12
14.39	0.0289	50.12
14.50	0.0065	31.62
14.78	0.0067	12.59
